# Could different direct restoration techniques affect interfacial gap and fracture resistance of endodontically treated anterior teeth?

**DOI:** 10.1007/s00784-021-03902-y

**Published:** 2021-04-15

**Authors:** Allegra Comba, Andrea Baldi, Carlo Massimo Saratti, Giovanni Tommaso Rocca, Carlos Rocha Gomes Torres, Gabriel Kalil Rocha Pereira, Felipe Luiz Valandro, Nicola Scotti

**Affiliations:** 1 Department Surgical Sciences, Dental School, University of Turin, Turin, Italy; 2 Department of Surgical Sciences, Dental School, University of Turin, Turin, Italy; 3 Division of Cariology and Endodontology, School of Dentistry, University of Geneva, Geneva, Switzerland; 4 Institute of Science and Technology, Department of Restorative Dentistry, São Paulo State University-UNESP, São José dos Campos, SP Brazil; 5 Department of Restorative Dentistry, Division of Prosthodontics, Federal University of Santa Maria, Santa Maria, Brazil; 6 Department of Surgical Sciences, Dental School, University of Turin, Turin, Italy

**Keywords:** 3D interfacial gap, Micro-CT, Fracture resistance, Fracture pattern, Post, Endodontically treated teeth

## Abstract

**Objectives:**

To evaluate different direct restoration techniques on various cavity designs in anterior endodontically treated teeth (ETT).

**Materials and methods:**

Ninety upper central incisors (*n* = 90) were selected, endodontically treated, and divided into three groups (*n* = 30) accordingly to the cavity design: minimal endodontic cavity access (group A), endodontic access + mesial class III cavity (group B), and endodontic access + two class III cavities (group C). Three subgroups (*n* = 10) were then created accordingly to the restoration technique: nano hybrid composite restoration (subgroup a), glass fiber post + dual-cure luting cement (subgroup b), and bundled glass fiber + dual-cure luting cement (subgroup c). Samples underwent micro-CT scan, chewing simulation, and a second micro-CT scan. 3D quantification (mm^3^) of interfacial gap progression was performed; then, samples underwent fracture resistance test. Data were statistically analyzed setting significance at *p* < 0.05.

**Results:**

Groups A and B showed significantly lower interfacial gap progression compared with group C. Subgroup b performed significantly better compared with subgroups a and c. Improved fracture strength was reported for group C compared with group A, while both subgroups b and c performed better than subgroup a.

**Conclusions:**

Cavity design significantly influenced interfacial gap progression and fracture resistance. Fiber posts significantly lowered gap progression and improved fracture resistance while bundled fibers only increased fracture resistance. A significant reduction of non-repairable fractures was recorded when fibers were applied.

**Clinical relevance:**

A minimally invasive approach, conserving marginal crests, should be applied whenever possible. Inserting a fiber post is indicated when restoring anterior ETT, in order to reduce gap progression, improve fracture resistance, and avoid catastrophic failures.

## Introduction

Restoration of endodontically treated teeth (ETT) remains a challenge for dental clinicians, as the endodontic treatment weakens the tooth structure in terms of biomechanical behavior compared with the vital counterpart. In fact, ETT are more brittle due to structural changes in dentin, loss of water, and weakened collagen cross-linking [1]. These changes lead to increased cuspal deflection during function, with consequent higher occurrence of fractures [2, 3]. For this reason, post-endodontic restoration challenge is to recover the biomechanical behavior of the tooth and prevent catastrophic fractures.

Several types of restorations have been proposed in literature to restore and reinforce ETT. In the past, traditional full coverage crowns in combination to metal post showed enhanced longevity, in the expense of an invasive procedure [4–6]. Thanks to the introduction of adhesive techniques, less invasive procedures are nowadays available to restore compromised teeth. Recent studies reported that the mechanical resistance and the longevity of ETT directly depend on the amount of residual tooth structure, meaning that a minimally invasive approach should be applied whenever possible. Direct resin composite restorations represent the least invasive approach in order to preserve the much sound structure possible. For this reason, they have been frequently studied to evaluate their efficacy when restoring an ETT, showing a significant increase in fracture resistance when the direct restoration was reinforced by fiber posts [7–10]. This trend was also confirmed by the in vivo evidence that highlighted a positive correlation between post-insertion and restoration longevity [11–13]. However, despite a great evidence regarding posterior teeth, few information concerning the direct restoration efficacy in endodontically treated anterior teeth is available.

In addition to the previously introduced concepts, it has to be considered that anterior restorations are subjected to high masticatory loads and parafunctional forces. Thus, fracture is a relatively common clinical failure that occur over time [14, 15]. A recent review by Heintze et al*.* reported that the lack of mechanical retention in class IV restoration must be considered an adhesive challenge and seems to lead to twice as high failure rate than class III restorations. A higher prevalence of failure in class IV restorations in bruxers was also reported by van Dijken et al. [16], showing that overloading and increased mechanical stresses in the restorations are making them more prone to fracture and secondary caries.

The evaluation of a direct restoration efficacy should not be focused on the tooth structure reinforcement effect only. Indeed, occlusal stresses generated during mastication and, especially, during parafunctional activities, such as bruxism, were shown to have a deleterious effect on the marginal adaptation of composites [17]. These mechanical stresses repeated over time lead to fatigue weakening of the adhesive interface, ultimately generating a gap that may further lead to microleakage [18]. Even if a direct correlation between microleakage and clinical parameters has not been proved [19], gaps that exceed a width of 60 μm could possibly lead to bacteria accumulation, ultimately leading to sensitivity and increased chance of secondary caries [20–22].

The aim of the present in vitro study was to evaluate the effect of different direct restoration techniques on endodontically treated anterior teeth with different cavity designs, analyzing interfacial gap and fracture resistance. The null hypothesis tested was that the cavity design (1) and the restoration technique (2) do not affect the interfacial gap and the fracture resistance of endodontically treated central incisors.

## Materials and methods

### Sample preparation

Ninety upper central incisors (*n* = 90) with similar crown and root size (length > 14 ± 2 mm), extracted within 4 months for periodontal reasons, were selected. Manual scaling was performed for surface debridement, followed by cleaning with a rubber cup and pumice. Specimens were disinfected in 0.5% chloramine for 48 h and then stored in 4% thymol solution at room temperature until use. Samples were double-checked with optical 4.5× magnification to exclude teeth with caries, previous restorations and visible cracks.

Selected teeth were endodontically treated using Pathfiles and ProTaper Next (Dentsply Maillefer, Ballaigues, Switzerland) to the working length, set at 1 mm short of the visible apical foramen. Irrigation was performed with 5% NaOCl (Niclor 5, Ogna, Muggiò, Italy) alternated with 10% EDTA (Tubuliclean, Ogna, Muggiò, Italy). The root canals were filled with gutta-percha cones trough a warm vertical condensation technique.

Specimens were then divided into three groups (*n* = 30 each) accordingly to the cavity design, which were performed by the same experienced operator.

- Group A: specimens exclusively presented a minimal endodontic cavity access at the cingulum level. Gutta-percha was removed up to 3 mm below the cemento-enamel junction (CEJ).
- Group B: additionally to the cavity access, a single class III cavity was prepared on the mesial side using an egg-shaped diamond bur. To ensure reproducible cavity dimensions as much as possible, the mesio-distal, linguo-buccal, and cervical-incisal extents of the tooth crown were measured with a caliper. Class III cavities included one third of the mesio-distal and linguo-buccal lengths and one quarter of the cervical-incisal extent. The cervical margin of the cavity was performed in enamel, ensuring a distance to the CEJ of 1 mm. Due to the selected mesio-distal dimension, the median part of the cavity was always connected to the endodontic cavity access.
- Group C: same as group B, but two class III cavities were prepared on mesial and distal side of each sample.

After cavity preparation, specimens were divided into three subgroups accordingly to the employed restoration technique (*n* = 10 each):

- Subgroup a: Cavity was etched with phosphoric acid (Conditioner 36, Dentsply, Konstanz, Germany) for 15 s, rinsed with water, and air-dried. A universal adhesive (Futurabond U, Voco, Cuxhaven, Germany) was applied uniformly at all cavity surfaces for 20 s using a micro brush, air-dried for 5 s, and light-cured for 20 s with a multiLED lamp (1400 mW/cm^2^; Bluephase Style, Ivoclar, Schaan, Luxembourg). A direct restoration with nano hybrid composite (Filtek Supreme XTE, 3M) was performed applying 2-mm-thick layers with horizontal layering technique.
- Subgroup b: Post-space was prepared with dedicated drills for a total of 8 mm depth (Rebilda Post Drill, diameter 1.2 mm). A dedicated fiber post (Rebilda Post, Voco) was luted with a dual-cure luting cement (Rebilda GT, Voco) following manufacturer instruction. After light-curing for 40 s with a multiLED lamp (1400 mW/cm^2^; Bluephase Style, Ivoclar), a direct composite restoration was performed as described for subgroup a.
- Subgroup c: Same as subgroup b, but using a bundled glass-fiber-reinforced composite post (Rebilda Post GT, Voco).

**Fig. 1:**
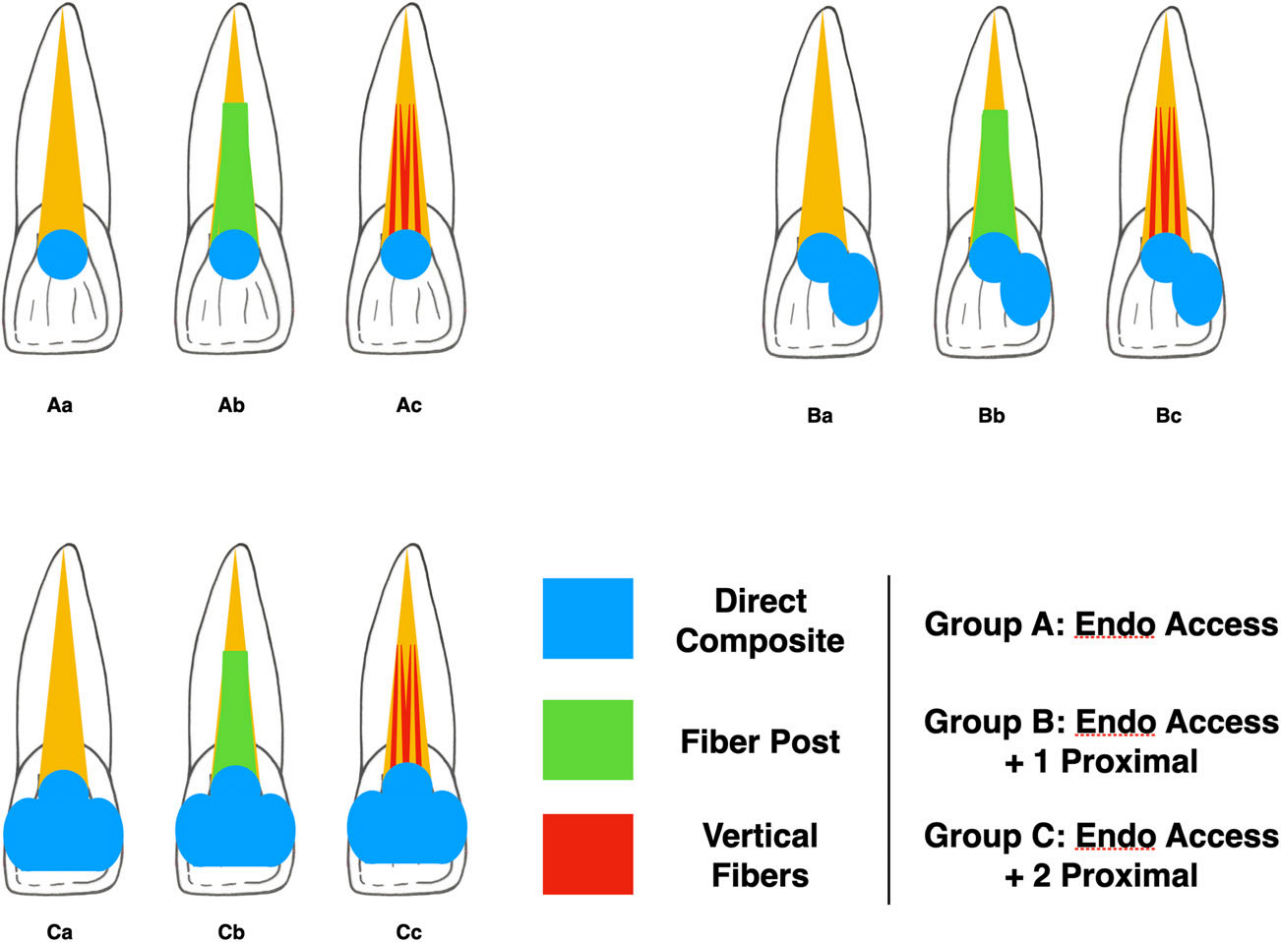
Schematic representation of the present study sample preparation protocol

All the restored specimens were finished and polished with fine-grit diamond burs and silicon points in order to obtain a smooth surface without over or under contouring, and then stored in distilled water. Figure 1 schematically reports the study design.

### Micro-CT analysis and cyclic fatigue test

Each sample underwent a micro-computed tomography (micro-CT) scan (SkyScan 1172; Bruker, Billerica, MA, USA) to evaluate interfacial gap. High-resolution scans were performed using the following parameters: voltage = 100 kV, current = 100 μA, aluminum and copper (Al + Cu) filter, pixel size = 15 μm, averaging = 5, rotation step = 0.5°. Images were reconstructed though NRecon software (Bruker, Billerica, MA, USA) in order to obtain DICOM files, with standardized parameters: beam hardening correction = 20%, smoothing = 3, ring artifact reduction = 9.

A CS-4.4 chewing simulator (SD Mechatronik; Feldkirchen- Westerham, Germany) was used for mechanical aging of the specimens. A 4-mm diameter metal cone was employed, using the following parameters: occlusal load = 50 N, frequency = 1 Hz, downward speed = 16 mm/s, and 2 mm sliding movement. The movement pattern was set from the palatal cingulum towards the incisal edge. The test was performed for 500,000 cycles in water at room temperature.

**Fig. 2:**
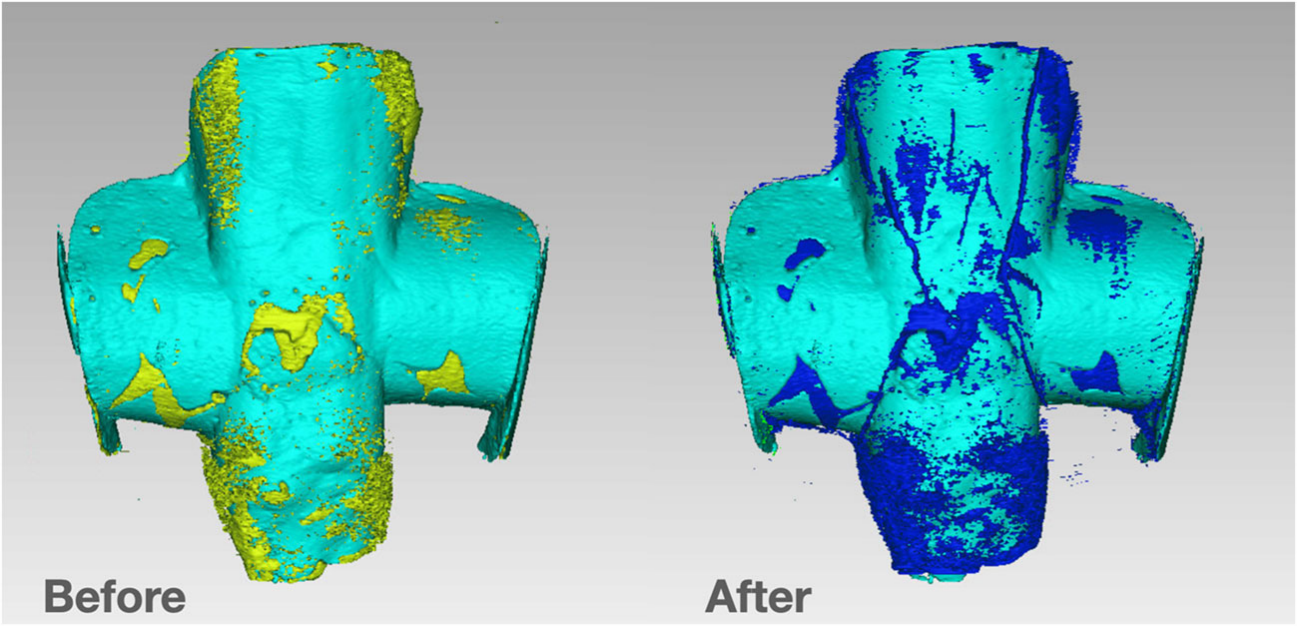
Random sample before (left) and after (right) chewing simulation. Light blue volume represents the restoration, seen from the inner surface. Yellow volume represents baseline void volume, while blue volume represents final void volume after cyclic fatigue. It is noticeable that many areas underwent degradation due to mechanical stresses and crack lines appeared. To specifically analyze interfacial gap progression, final data recorded consisted in blue volume minus yellow volume

To reveal interfacial gap progression between the restoration and the tooth structure after cyclic fatigue, samples were subjected to a second scan with same baseline parameters to ensure consistency in the grayscale values. Initial scans were then reconstructed with NRecon using the same protocol and aligned with post-chewing scans using DataViewer TM software (Bruker, Billerica, MA, USA). Thresholding was performed automatically with Mimics Medical 20.0 software (Materialise, Ann Arbor, MI, USA), in order to obtain a void mask representing gaps and voids inside the tooth-restoration complex, with external boundaries set at 1 mm from the direct restoration. A Hounsfield unit (HU) range of 1024 to 950 was selected to maximize void visualization. The volume of the mask was automatically calculated by the software and recorded in mm^3^. In order to specifically analyze gap progression and exclude composite internal bubble volume, the result obtained from the baseline scan was subtracted from the volume of the second scan. Figure 2 reports the 3D rendering of a random sample (restoration and voids), seen from the inner surface (in contact with the tooth), before and after chewing simulation.

### Fracture resistance test and failure mode analysis

Samples were then submitted to a static fracture resistance test using a universal testing machine (Instron 10-S; Canton, MA, USA) with a 4-mm diameter metal cone crosshead welded to a tapered shaft and applied to the sample at a constant speed of 0.5 mm/min and an angle of 30° to the long axis of the tooth. Load was applied on the palatal cingulum until fracture and the maximum breaking loads were recorded in Newton (N).

**Fig. 3:**
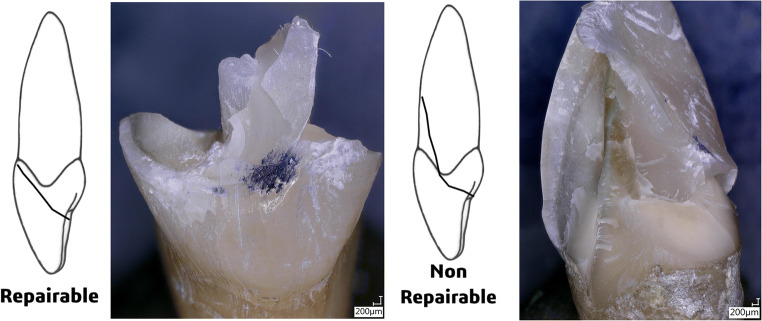
Random fractures recorded among samples. Notice how CEJ was taken as a reference point to distinguish reparable and non-reparable fractures

Broken specimens were analyzed under a stereomicroscope (SZX9; Olympus Optical Co., Ltd., Tokyo, Japan). The types of failure were determined and compared, distinguishing between catastrophic fractures (non-reparable, below the CEJ) and non-catastrophic fractures (reparable, above the CEJ). Figure 3 reports two different fractures, as well as a schematic representation for clarification.

### Statistical analysis

To examine the effects of the factors “cavity design” and “restoration technique” on interfacial gap progression and the fracture resistance, a two-way analysis of variance test (ANOVA) was conducted. Post hoc pairwise comparison was performed using Tukey test. All statistical analyses were performed using STATA software (ver. 12.0; StataCorp, College Station, TX, USA).

## Results

Interfacial gap progression data, expressed as means ± standard deviation in mm^3^, and fracture resistance, expressed in N, are summarized, respectively, in Table 1 and Table 2. Two-way ANOVA showed that interfacial gap was significantly related to the cavity design (*p* < 0.001) as well as to the restoration technique (*p* < 0.001), as well as the interaction between the two factors (*p* < 0.001). Tukey post hoc test revealed that groups A and B showed significantly lower interfacial gap increase after cyclic fatigue compared with group C and subgroup b showed significantly reduced gap formation compared with subgroups a and c.

**Table 1:** Mean interfacial gap variations ± standard deviation, expressed as mm^3^, for each group and subgroup

	Subgroup a (no post)	Subgroup b (fiber post)	Subgroup c (bundled fibers)
Group A (endodontical access)	0.12 ± 0.06	0.29 ± 0.09	0.27 ± 0.08
Group B (mesial class III cavity)	0.27 ± 0.09	0.19 ± 0.08	0.22 ± 0.07
Group C (mesial and distal class III cavities)	0.67 ± 0.19	0.35 ± 0.10	0.48 ± 0.15

**Table 2:** Mean fracture resistance ± standard deviation, expressed in Newton (N), for each group and subgroup

	Subgroup a (no post)	Subgroup b (fiber post)	Subgroup c (bundled fibers)
Group A (endodontical access)	542.6 ± 207.2	667.2 ± 243.3	660.4 ± 231.7
Group B (mesial class III cavity)	507.7 ± 143.1	718.7 ± 149.7	643.6 ± 208.8
Group C (mesial and distal class III cavities)	335.8 ± 86.5	663.1 ± 166.3	537.8 ± 108.2

Concerning fracture resistance, two-way ANOVA showed a significance difference both for the factor “cavity design” (*p* = 0.023) and for the factor “restoration technique” (*p* < 0.001). The Tukey post hoc test highlighted statistical improved fracture strength for subgroup b (*p* < 0.001) and c (*p* = 0.005) compared with the subgroup a. Concerning the cavity design factor, Tukey test showed statistical significance when group C was compared with group A (*p* = 0.005), with group C performing significantly worse (lower fracture resistance). Recorded fracture patterns, classified between repairable and non-repairable, are reported in Table 3.

**Table 3:** Fracture patterns for each group and subgroup, divided between repairable (rep) and non-repairable (non-rep)

	Subgroup a (no post)		Subgroup b (fiber post)		Subgroup c (bundled fibers)	
	Rep	Non-rep	Rep	Non-rep	Rep	Non-rep
Group A (endodontical access)	2	8	8	2	7	3
Group B (mesial class III cavity)	2	8	8	2	8	2
Group C (mesial and distal class III cavities)	0	10	7	3	5	5

**Fig. 4:**
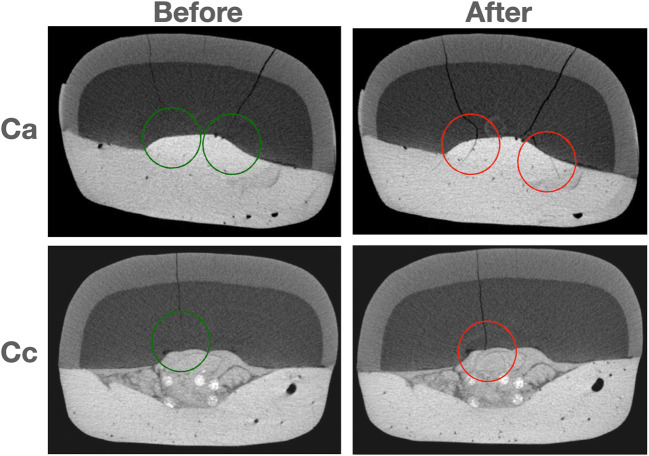
The first row shows a random sample (Ca) before and after chewing simulation. It is noticeable that cracks propagated from the tooth structure to the buildup itself. The second row shows another sample reinforced with fibers (Cc), where the crack propagation is clearly limited to the tooth structure

After an accurate analysis of the reconstructed images, it was also observed, from a qualitative point of view, that some of the samples randomly presented pre-existent micro-cracks, not visible at 4.5× magnification, which propagated as a consequence of chewing simulation. Micro-cracks showed a tendency to continue inside the composite buildup when no fibers were applied (subgroup a) compared with samples reinforced with fibers (subgroups b and c). Figure 4 illustrates an example of this trend, showing the propagation of initial micro-cracks in two random samples from subgroups a and c, before and after chewing simulation.

## Discussion

Clinical studies already demonstrated that incisors and canines have an overall higher failure rate compared with posterior teeth, as the occlusal forces are more transverse [23, 24]. The cyclic fatigue derived from chewing, especially transversal forces, causes a progressive degradation and therefore “opening” of the adhesive interface [17, 18]. The consequent marginal leakage is of critical concern when referring to composite restorations since it might lead to secondary caries and cracks, letting the tooth more prone to fracture [20, 21]. Moreover, in ETT, marginal leakage led to a potential bacterial recolonization of the root canal system, ultimately causing endodontic failure [25].

Basaran et al. showed that a percentage of dye leakage at the interface between the post and the root canal was always present, regardless of the fiber post or the adhesive technique employed [26]. However, to date, two-dimensional techniques for the analysis of the interfaces are to be considered obsolete and limited compared with three-dimensional investigation methods. A recent technique to detect interfacial gaps is represented by μCT, which allows, without destroying the specimen, to generate 3D images. The number of studies using μCT in restorative dentistry is increasing, as this technique has proved effective for the evaluation of internal adaptation of composite resin restoration [27–32]. In the present study, cyclic intermittent loading induced an interfacial gap opening in all specimens, corroborating in vivo and in vitro previous findings that showed functional and parafunctional stresses, especially transversal forces, are able to cause marginal gap opening on adhesive interfaces [17, 18].

Based on the present study results, the cavity extension as well as the use of fiber post were crucial in reducing the interfacial gap progression after cyclic fatigue; thus, the first null hypothesis was rejected. Interfacial gap openings occur during fatigue when cyclic forces induce a tooth flexion which is higher in non-vital teeth due to their reduced stiffness [33]. Loss of tooth structure is a key factor for stress resistance of endodontically treated teeth, in anterior as well as in posterior teeth. As demonstrated by Reeh et al. referring to premolars, the loss of marginal ridges can lead to a diminished fracture resistance going from 44 to 66% [33]. Obviously, if more tooth structure is preserved, cyclic forces find a higher resistance to flexion, thus leading to less interfacial gap formation. The present study showed that the loss of one or two marginal ridges is immediately correlated to increased interfacial gap, because they represent the anatomical portion in anterior teeth that provides resistance to traversal loads. The use of a fiber post is indeed crucial when extended cavities are present as their mechanical properties are close to the dentin [34, 35]. Consequently, they can reproduce the natural load transmission mechanisms to the tooth structure reducing the risk and entity of gap formation. Moreover, an increased flexural strength when using a fiber post compared with composite-only build-up has already been demonstrated by several authors [36]. The higher flexural strength of fiber post might mediate loads between dentin and restoration materials, therefore resulting in a more homogenous stress distribution [37]. On the other hand, the placement of vertical bundled glass fibers within the root canal did not significantly reduce the gap increase during cyclic fatigue, probably due to the lower flexural strength of this restorative solution if compared with the traditional glass fiber post.

Possible ways to restore compromised ETT were studied and analyzed in the past by many authors [4, 38, 39], who demonstrated an important reduction in tooth fracture when a full coverage crown was performed. However, this option is very demanding in terms of economical and biological costs for the patient. This concept is particularly true when referring to anterior teeth, whose fracture resistance is similarly correlated to the presence of residual tooth structure [40, 41], but it is subjected to different biomechanical stresses during function and parafunction.

The present study results clearly showed that the cavity configuration in anterior teeth is directly correlated to the fracture resistance, which could be partially recovered by using a fiber-supported composite restoration. Thus, the second null hypothesis was rejected. It has been recently suggested, in order to improve fracture resistance in ETT, to insert fibers within direct resin composite restorations [42, 43]. Thanks to their elastic modulus similar to dentin and stress bearing capabilities, fibers might reinforce the structure and lead to fewer root fractures. Literature, however, is not unanimous about the usage of fibers, with studies affirming that there is no significant difference in the use of a classic composite build-up and its corresponding post system [44]. On the other hand, other authors affirm that for anterior ETT, fiber post-placement seems advisable to improve static load resistance, especially in cases with extensive loss of coronal tissues [45]. This is in accordance with the present study results, which reported ETT performing significantly better in fracture resistance test when a post or vertical bundled fibers were used. This reinforcement effect was mainly advisable in group C, probably because buccal enamel, incisal margin, and oral cingulum are less involved in the tooth structure reinforcement compared with proximal ridges. This could also explain the different results obtained by Lausnitz et al.: a less invasive cavity design surely helps the specimen in resisting both fatigue cycles and fracture loads [46]. This is also accordance with the results of Vadini et al. that reported a significant benefit in resistance to static loads when a post was placed, particularly in cavity designs with extensive loss of coronal tissues (two class II cavities) [45]. Anyway, further studies should focus and evaluate the contribution to the resistance to occlusal loads of the anatomical components of the anterior teeth in order to better understand the impact of cavity configuration and extension on their resistance to fatigue phenomena.

As demonstrated by Newman et al. [47], fiber-supported composite appears to dissipate forces along the root canal system, reducing peak stresses on the root and therefore moving the critical fracture point coronally, ultimately leading to repairable fractures [48, 49]. On the other hand, rigid posts such as carbon fiber or cast posts and core seem to be more prone to cause non-repairable root fractures due to their elastic modulus [50]. Hayashi et al. studied the fracture mode when teeth restored with different post system were subjected to oblique and vertical load, concluding that vertical loadings caused crack propagation in the middle and apical portion of the roots, while with oblique loads, most of the fractures occurred in the cervical part of the root when fiber posts were used, and in the middle part, when prefabricated metallic or cast metallic post-core were used [51]. Chieruzzi et al. showed that when a fiber-post is used, the stress generated through dentin, cement, and post is well-distributed and without any relevant peak. Therefore, it can be concluded that the use of glass fiber allows to simulate the mechanical behavior of natural tooth [52]. The fracture pattern analysis performed in the present study confirmed previous findings, as all samples restored with fiber posts showed more favorable fracture patterns. In this context, vertical bundled fibers showed better performance compared with a direct composite restoration, but inferior performances compared with fiber post-supported composite restoration, especially where an extensive loss of structure was simulated.

Lastly, in some sample, it has been noted that fiber seems to limit or avoid the propagation of micro-cracks, as previously shown in Fig. 4, ultimately acting as force-breakers. In most of the samples of subgroup a (no post), the propagation of dentinal cracks, which were randomly present before cyclic fatigue test, continued in the composite restoration, while in subgroups b and c, fibers were able to block or reduce this trend. This data could be important to understand the resistance to cyclic loads, even considering that the majority of dental restorations fail under subcritical, cyclic occlusal loads over an extended period of time, during which the interfacial bond degrades progressively.

## Conclusions

Based on the obtained results, it can be concluded that:

- Cavity design significantly influences interfacial gap progression, fracture resistance, and fracture pattern.
- Fiber post-supported composite significantly reduced gap progression and improved fracture resistance of ETT anterior teeth. Thus, the insertion of a fiber post is indicated, even to improve the probability of a favorable fracture pattern.
- Vertical bundled fibers were not able to reduce interfacial gap progression significantly, but they increased fracture resistance and slightly improved fracture pattern, even if not as much as conventional fiber post.

Further in vitro studies are necessary to evaluate the crack propagation during fatigue.
